# Nasopharyngeal microbiome composition associated with *Streptococcus pneumoniae* colonization suggests a protective role of *Corynebacterium* in young children

**DOI:** 10.1371/journal.pone.0257207

**Published:** 2021-09-16

**Authors:** Lei Xu, Joshua Earl, Michael E. Pichichero

**Affiliations:** 1 Center for Infectious Diseases and Immunology, Research Institute, Rochester General Hospital, Rochester, New York, United States of America; 2 Department of Microbiology & Immunology, Centers for Genomic Sciences and Advanced Microbial Processing, Drexel University College of Medicine, Philadelphia, Pennsylvania, United States of America; Defense Threat Reduction Agency, UNITED STATES

## Abstract

*Streptococcus pneumoniae* (Spn) is a leading respiratory tract pathogen that colonizes the nasopharynx (NP) through adhesion to epithelial cells and immune evasion. Spn actively interacts with other microbiota in NP but the nature of these interactions are incompletely understood. Using 16S rRNA gene sequencing, we analyzed the microbiota composition in the NP of children with or without Spn colonization. 96 children were included in the study cohort. 74 NP samples were analyzed when children were 6 months old and 85 NP samples were analyzed when children were 12 months old. We found several genera that correlated negatively or positively with Spn colonization, and some of these correlations appeared to be influenced by daycare attendance or other confounding factors such as upper respiratory infection (URI) or *Moraxella* colonization. Among these genera, *Corynebacterium* showed a consistent inverse relationship with Spn colonization with little influence by daycare attendance or other factors. We isolated *Corynebacterium propinquum* and *C*. *pseudodiphtheriticum* and found that both inhibited the growth of Spn serotype 22F strain *in vitro*.

## Introduction

*Streptococcus pneumoniae* (Spn) causes a variety of illnesses, including pneumonia, otitis media, bacteremia, and meningitis [1, 2]. Despite availability of pneumococcal vaccines, Spn remains the most common cause of bacterial infection in the developing world and most frequently infects children under 5 years old or elderly over 65. It was included as one of 12 priority pathogens by WHO in 2017 [3]. Spn colonizes the nasopharynx (NP) of children in the first month of life and 27–65% of children carry Spn asymptomatically [3]. It is incompletely understood how Spn progresses from a commensal state to a pathogenic state and eventually invades tissues and blood stream to cause local invasiveness and systemic infections. We hypothesize that commensal bacteria in NP may influence this process and promote or prevent Spn-related diseases in children.

The NP microenvironment harbors commensal flora, which maintain immune homeostasis and suppress pathogenic progression and/or colonization of respiratory tract pathobionts. Based on studies in the gut and in the NP, protective microbiota compete with pathogens in the microenvironment, through nutrient deprivation, production of anti-microbial molecules, and modulating the innate and adaptive immune system [3–12]. One dominant commensal genus in the NP is *Corynebacterium*. There are over 100 species of *Corynebacterium* [13], and at least 23 are present in NP [14]. Whether the different species exert distinct effects on Spn pathogenesis is not clear, highlighting the limit of our understanding of the influences of *Corynebacterium* on Spn-related illnesses. An inverse correlation of *Corynebacterium* detection in the NP to Spn colonization had been reported [15–18]. Some *Corynebacterium* spp. showed direct inhibition on *in vitro* Spn growth or *in vivo* colonization. For example, *C*. *accolens* was shown to inhibit growth of Spn *in vitro* [15]. *C*. *pseudodiphtheriticum* was reported to prevent *Spn*-induced pneumonia in mice but this effect appeared to be specific to particular *C*. *pseudodiphtheriticum* strains [19, 20].

In this study, we examined the NP microbiome of children at age 6 and 12 months with or without Spn colonization detected by bacterial culture. We found that detection of *Corynebacterium* spp. was inversely correlated with Spn colonization, consistent with previous reports [15–18]. We isolated two species of *Corynebacterium*: *C*. *pseudodiphtheriticum* and *C*. *propinquum*. We then investigated the effects of these *Corynebacterium* spp.on Spn growth *in vitro* and found that both exhibited inhibitory functions.

## Materials and methods

The Rochester General Hospital IRB approved the study and written informed consent was obtained from parents before enrollment. All methods were performed in accordance with the IRB’s relevant guidelines and regulations.

### Subject information and sample collection

NP washes were obtained during a prospective cohort study conducted in Rochester NY from 2006–2018 involving healthy children of 6 and 12 months old for study of respiratory illnesses, in particular acute otitis media (AOM), supported by the National Institutes of Deafness and Communication Disorders. Details of the cohort have been described previously [21–23]. Briefly, these children were recruited from middle-class suburban sociodemographic pediatric practices in Rochester, NY. Informed consent was obtained in writing at enrollment from the child’s parents or legally authorized representative. The parent/guardian agreed to provide follow-up information and arrange for all scheduled visits. Because this study was designed to investigate the impact of microbiome on AOM in children, individuals with uncertain diagnosis of AOM were excluded from the study. Additional exclusion criteria included diagnosis of otorrhea, presence of tympanostomy tube, or diagnosis of Down syndrome, cleft palate, craniofacial disorders, cystic fibrosis/mucoviscidosis immotile cilia syndrome, congenital immunodeficiency or HIV/AIDS, or other medical conditions that may interfere with implementation of the protocol or interpretation of study results. All children were vaccinated with pneumococcal conjugate vaccines (either PCV13 or PCV7 according to availability).

Definitions: Viral URI: Diagnosis of viral URI was made when children presented symptoms of nasal congestion, rhinorrhea, cough, and/or sore throat with or without fever, following established guidelines [24–27]. AOM: AOM was diagnosed using pneumatic otoscopy by validated otoscopists according to American Academy of Pediatrics guidelines [28]. The children presented acute onset of symptoms consistent with AOM and had tympanic membranes (TMs) that were: 1) bulging or full, with a cloudy or purulent effusion, or 2) completely opacified, and 3) with reduced or absent mobility.

NP washes from the children were cultured *in vitro* on Chocolate agar and tryptic soy agar (TSA) with 5% sheep blood (BD) for identification of Spn and other common respiratory bacterial pathogens using microbiology methods previously described [29, 30]. Spn was identified based on their colony morphology, alpha hemolytic activity on TSA blood plates, and sensitivity to optochin disc. *Haemophilus influenzae* (Hi) was identified based on their colony morphology, gram negative staining, growth on Chocolate agar plates but not on TSA blood plates, and their presence or absence of growth in specific quadrants on hemo ID quad plates (BD), depending on X- and V-factor requirements for the various *Haemophilus* species. We did not confirm every Hi isolates but our recent study found that more than 95% of Hi strains were non-typable (Fuji N et al, in press). *Moraxella catarrahlis* (Mcat) was identified based on colony morphology, gram-negative stain, positive oxidase reactivity, and positive reactivity to Remel Catarrahlis Test disc (Thermofisher). *Staphylococcus aureus* (SA) was identified based on its beta-hemolytic activity on blood plates and its being coagulase-positive and catalase-negative. Based on these microbiology methods, samples were split into Spn+ or Spn- groups for microbiome comparisons. The demographic information of the patient samples is listed in Table 1. The samples were stored at -80°C in Virus Transfer Media (VTM) after collection and then sent for 16S rRNA gene sequence analysis at the Microbiome Core Facility, University of North Carolina, Chapel Hill (https://www.med.unc.edu/microbiome/). All the above information on the samples could be accessed in S1 Table.

**Table 1:** Demographic factors associated with Spn colonization.

	# of samples	Race (White: non-white)	Female: Male	Breast-feeding (Yes:No)	Exposure to smoke (Yes:No)	Atopy (Yes:No)	Abx Treatment (Yes:No)	Daycare Attendance (Yes:No)	Siblings (Yes:No)
Spn+									
6 m	25	22:3 [0.53]	10:15 [0.46]	10:15 [0.46]	0:24 [0.17]	5:18 [0.57]	1:20 [0.41]	9:15 [0.0006]**	3:22 [1]
12 m	28	24:4 [0.77]	12:16 [0.17]	10:16 [0.47]	1:26 [0.26]	6:19 [0.60]	8:13 [0.060]	10:17 [0.0078]*	4:24 [1]
**total**	**53**								
Spn-									
6 m	48	39:9 [0.53]	24:24 [0.46]	24:23 [0.46]	6:42 [0.17]	13:29 [0.57]	6:35 [0.41]	2:45 [0.0006]**	6:42 [1]
12 m	56	46:10 [0.77]	33:23 [0.17]	27:28 [0.47]	8:47 [0.26]	15:34 [0.60]	7:38 [0.060]	6:49 [0.0078]*	9:47 [1]
**total**	**104**								

Note: Demographic factors are listed as column names. The number of children who were female or male, or who were Yes or No for a particular demographic factor is shown for each age group (6-month or 12-month) and for each Spn colonization phenotype (Spn+ or Spn-). In each age group, the proportion of children carrying a demographic trait was compared between Spn+ and Spn- samples and statistical significance assessed by Fisher’s Exact test. The *p* values are listed in square brackets.

*: *p* < 0.05;

**: *p* < 0.005.

### 16S rRNA gene sequencing analyses

Sample submission and sequencing analyses have been previously described [23]. Briefly, the V4 region of 16S rRNA gene was sequenced via Illumina sequencing, which were processed by Illumina Bcl2Fastq 2.18.0.12 and Cutadapt, and DADA2 [31]. The fastq sequences were deposited at Sequence Read Archive (https://www.ncbi.nlm.nih.gov/sra) with accession number PRJNA720045. Bar plots were created by ggplot2 in R (version 3.6.1, r-project.org), after the conglomerate of data files at phylum, class, and family level. Alpha diversity indices of the samples were measured via estimate_richness and graphed by the plot_richness command in Phyloseq in R. To measure beta diversity of samples, Bray-Curtis distance and weighted unifrac between samples were calculated via phyloseq::distance command and NMDS plots were created by ordination method. For differential abundance analyses, 30 genera with the highest abundance, or genera present in at least 50% of the samples, were analyzed through DESeq2 (version 1.24.0) in R and the outcomes were corrected for batch effects. Adjusted *p* values were calculated after correction for multiple hypothesis testing using Holm-Bonferroni method and those less than 0.05 were graphed in ggplot2.

### Isolation of *Corynebacterium* species

Cultures from NP swabs were plated on blood agar plates. Colonies with an appearance consistent with *Corynebacterium* spp. were randomly selected for PCR analyses [32] and for culturing in brain heart infusion (BHI) media at 37°C. Primers targeting the *rpoB* gene were used in the PCR analyses, which effectively distinguish species in the *Corynebacterium* genus [33]. The primer sequences are: C2700F: 5’-CGTATGAACATCGGCCAGGT– 3’, and C3130R: 5’-TCCATTTCGCCGAAGCGCTG-3’. The PCR program used was: 95°C 5min then 40 cycles of 95°C 15s, 55°C 15s, 72°C 15s. PCR products were separated on 1.5% agarose gel with an expected size of ~450bp and were subsequently isolated and extracted for DNA sequencing. Sequencing results were searched against GenBank database (blast.ncbi.nlm.nih.gov/Blast.cgi) for matches. *C*. *propinquum* and *C*. *pseudodiphtheriticum* were scored as the top candidates.

### *In vitro* co-culture experiments

*C*. *propinquum* and *C*. *pseudodiphtheriticum* were cultured in BHI media to reach OD_600_ = 0.5. 5 μl of the culture was spotted on a blood agar plate and grown at 37°C for one day (for *C*. *propinquum*) or two days (for *C*. *pseudodiphtheriticum*), before 5 μl of Spn 22F strain (grown in THBY media to OD_600_ = 0.5) was spotted next to the *Corynebacterium* at different distances. Images were taken every 24 hours and analyzed by ImageJ. To correct for the variations in images taken on different days, the diameter of each image for the same plate was measured on each day and the ratio of its square over the square of the diameter measured on the first day was used as a normalizing factor. The area covered by each colony was measured via ImageJ, which was divided by the normalizing factor before being imported for graphing in Microsoft Excel. In the second approach to visualize how *Corynebacterium* might affect Spn growth, 600 μl of Spn 22F (OD_600_ = 0.5) or non-Spn alpha-hemolytic *Streptococcus* (AHS) as a control was spread onto 10 cm blood agar plates to form a lawn. The AHS strain was an alpha-hemolytic *Streptococcus* isolate from a pediatric patient that showed resistance to optochin, in contrast to *Streptococcus pneumoniae*. Cultures of *C*. *propinquum* and *C*. *pseudodiphtheriticum* were concentrated by centrifugation. 5 μl of the pellet was spotted onto the Spn22F or AHS lawn and incubated at 37°C. Images were taken the next day.

### Statistics

Demographic factors and other factors that may influence Spn colonization were compared between Spn+ and Spn- samples by Fisher’s Exact test (https://www.socscistatistics.com/tests/fisher/default2.aspx). The difference in abundance of taxa, as shown in bar plots and in alpha diversity between Spn+ and Spn- samples, was calculated by Wilcoxon signed-rank test with Holm-Bonferroni adjustment. Statistical significance of differences between two populations based on beta diversities was calculated by Permanova via Adonis in R.

## Results

### Demographic and risk factors that associate with Spn colonization

96 children were included in the study cohort. 73 NP samples were analyzed when children were 6 months old and 84 NP samples were analyzed when children were 12 months old (Table 1). Gender, race, breast-feeding history, exposure to smoke, history of atopy, antibiotic treatment (30 days prior to sample collection), daycare attendance, and presence of siblings were variables that had frequently been examined for their association with Spn colonization and some were reported as risk factors [34–39]. We evaluated these factors in our cohort. As previously reported [34], daycare attendance correlated significantly with Spn colonization in the child’s NP, but none of the other demographic factors did in this study cohort (Table 1).

We investigated the association between Spn colonization and common respiratory pathogens, clinically-diagnosed viral upper respiratory infection (URI), and proness to acute otitis media (AOM) (Table 2). Spn colonization in NP has been reported to positively correlate with colonization of other otopathogens and with URI [36, 40]. In our study, *Moraxella catarrhalis* detected by culture was significantly associated with Spn carriage at 6 months, although not at 12 months (Table 2). *Haemophilus influenzae* (Hi) did not show significant association with Spn colonization although our group has previously reported an association [40]. This lack of significance in association could be due to the fact that few samples were positive for Hi in this cohort. *Staphylococcus aureus* did not show correlation with Spn colonization in our cohort (Table 2), although a negative association had been reported in other studies [41, 42]. URI was significantly associated with Spn carriage in 12-month samples but not so in 6-month samples (Table 2). AOM is a common disease in children and is frequently caused by Spn infection [43]. We have previously reported that while some children never develop AOM (AOM-free), some have frequent occurrences [44]. Those who develop at least 3 episodes of AOM (confirmed by tympanocentesis) within 6 months or 4 episodes in 12 months were categorized as sOP (stringently-defined Otitise-Prone). Elevation of Spn colonization was reported in sOP children compared with AOM-free children [36]. Similarly, in our cohort, sOP children had more frequent Spn colonization at both 6 months and 12 months of age compared to AOM-free children (Table 2).

**Table 2:** Other factors associated with Spn colonization.

	# of samples	Mcat	Hi	SA	URI	AOM-free: sOP
Spn+						
6 m	26	15+, 11- [0.011]*	3+, 23- [0.69]	0+; 25- [0.088]	5+, 21- [0.76]	13:12 [0.036] *
12 m	29	10+, 19- [0.18]	3+, 26- [0.69]	1+, 27- [0.26]	10+,19- [0.024]*	16:12 [0.037]*
**total**	**55**					
Spn-						
6 m	48	12+, 36- [0.011]*	4+, 44- [0.69]	6+, 42- [0.088]	8+, 40- [0.76]	37:11 [0.036] *
12 m	56	11+, 45- [0.18]	4+, 52- [0.69]	8+, 48- [0.26]	7+, 48- [0.024] *	45:11 [0.037]*
**total**	**104**					

Note: Specific factors are listed as column names. The number of children who were positive (+) or negative (-) for each potential bacterial respiratory pathogen or for URI, or were AOM-free or sOP, are indicated for each age group (6-month or 12-month) and for each Spn colonization phenotype (Spn+ or Spn-). In each age group, the proportion of children colonized with an otopathogen or URI, or designated as AOM-free or sOP, was compared between Spn+ and Spn- samples and statistical significance was assessed by Fisher’s Exact test. The *p* values are included in square brackets. SA: *Staphylococcus aureus*.

*: *p* < 0.05.

### Microbiome composition during Spn colonization

**Fig 1:**
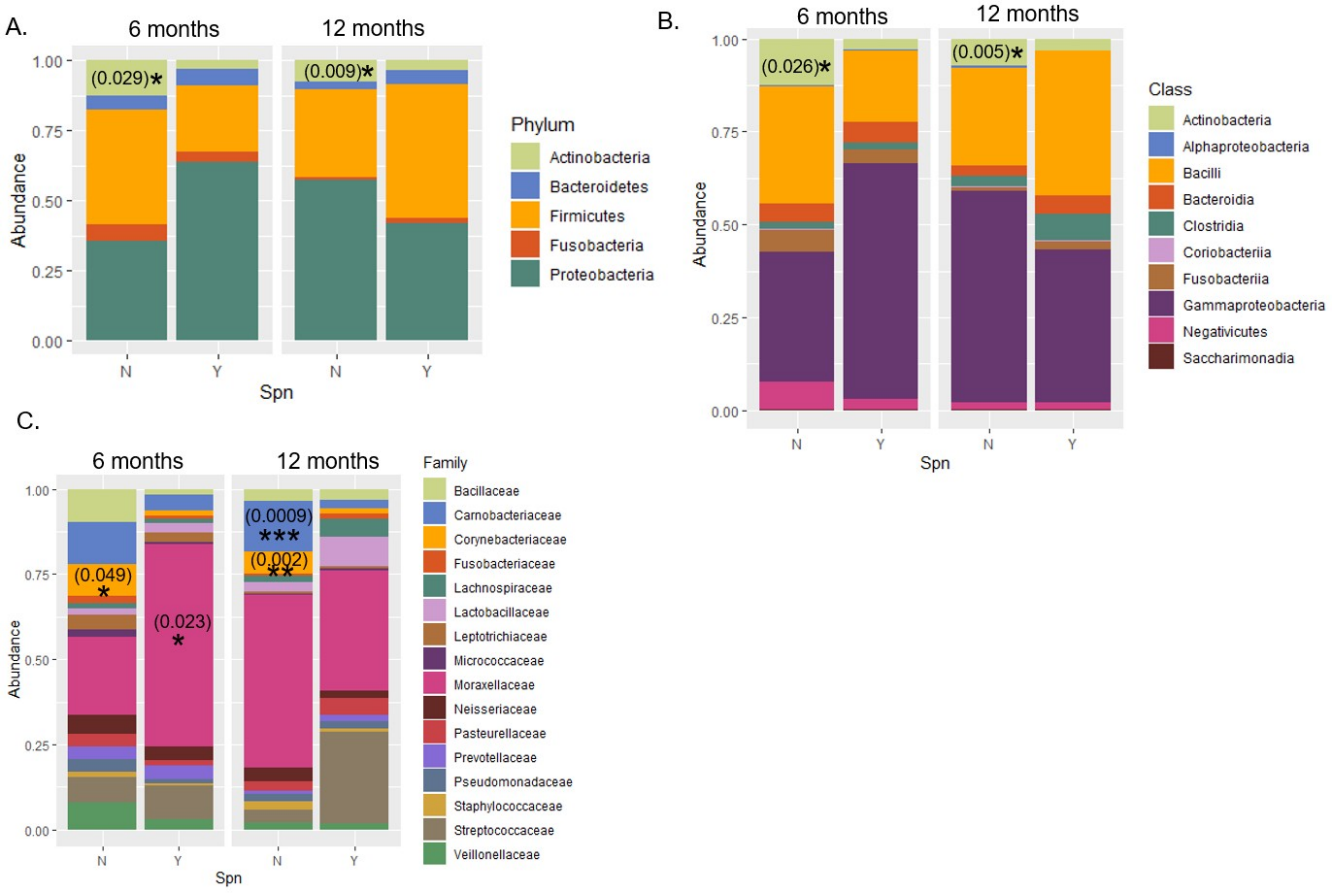
Abundance of taxa in Spn+ and Spn- NP samples. Proportion of taxa in Spn+ or Spn- NP samples at different ages were shown at the level of phylum (A), class (B), family (C) as bar plots. *: *p* < 0.05; **: *p* < 0.005; ***: *p* < 0.0005, Wilcoxon signed-rank test with Holm-Bonferroni adjustment. The absolute *p* values are included in parentheses on the graphs.

16S rRNA gene sequencing was performed to analyze the microbiome composition in each NP sample. Five phyla were most abundant: Actinobacteria, Bacteroidetes, Firmicutes, Fusobacteria, and Proteobacteria (Fig 1A). Among these, Actinobacteria was significantly reduced in Spn+ samples of both 6 (p = 0.029) and 12 months of ages (p = 0.009), relative to Spn- samples. This was also observed at the class level, where Actinobacteria was found inversely correlated with Spn colonization (Fig 1B; p = 0.026 in samples from 6-month olds and p = 0.005 in samples from 12-month olds). At the family level, *Corynebacteria*ceae was less abundant in Spn+ samples than in Spn- samples from children of 6 (p = 0.049) or 12 months old (p = 0.002), *Moraxellaceae* family was more abundant in Spn+ samples from 6 month old children (p = 0.023), and *Carnobacteriaceae* family was less abundant in Spn+ samples from 12 month old children (p = 0.0009).

**Fig 2:**
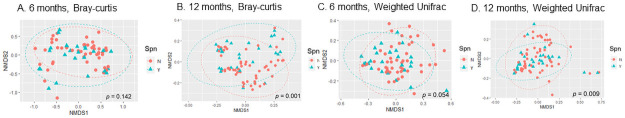
Difference in beta diversity of microbiome between Spn+ and Spn- NP samples in children at 12 months. Bray-Curtis distance (A and B) or weighted unifrac (C and D) of samples collected at 6 months or 12 months were measured and visualized on NMDS plots. Permanova was performed for samples stratified by age to assess the difference between Spn+ and Spn- samples. The *p* values are shown at the right bottom corner of each plot.

Four indices of alpha diversity were measured and no significant difference was observed between Spn+ and Spn- samples from children of either 6 or 12 months old (S1 Fig). In contrast, beta diversity measurements showed composition differences in the microbiome of Spn+ and Spn- samples. Bray-Curtis dissimilarity and weighted unifrac were measured among the samples and visualized in NMDS plots (Fig 2). Both measurements identified a significant difference in microbiome composition between Spn+ and Spn- samples from children at 12 months of age (p = 0.001 and 0.009), although only weighted unifrac identified a significant difference in microbiome composition between Spn+ and Spn- samples from 6-month old children (p = 0.054).

**Fig 3:**
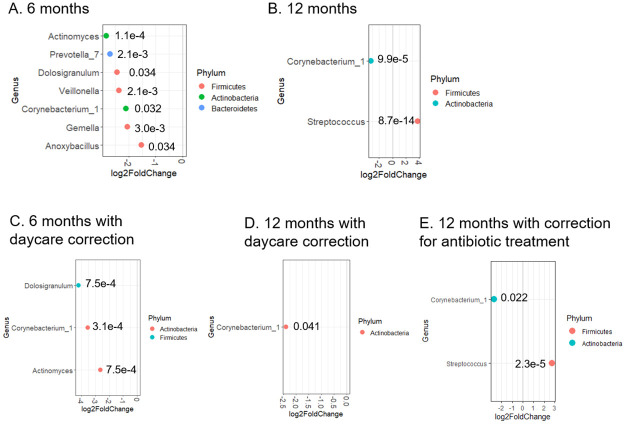
Bacterial genera that show differential abundance between Spn+ and Spn- samples. Abundance of bacterial genera was compared between Spn+ and Spn- samples collected at 6 months (A), or at 12 months (B) without correction for daycare attendance, or with correction for daycare attendance (C, 6 months; D, 12 months) using DESeq2 software (see Materials and methods). The ones with adjusted *p* value less than 0.05 are shown as dots in the graphs. The adjusted *p* value is indicated next to each dot. The x axis exhibits the log_2_ ratio between abundance of bacterial genera in Spn+ samples and that in Spn- samples.

Individual bacteria genera that change abundance upon Spn colonization were identified (Fig 3). In samples from 6 month-old children, reduced abundance of *Actinomyces* (p = 0.0001), *Prevotella_7* (p = 0.002), *Dolosigranulum* (p = 0.034), *Veillonella* (p = 0.002), *Corynebacterium_1* (p = 0.032), *Gemella* (p = 0.003), and *Anoxybacillus* (p = 0.034) were identified in Spn+ samples compared to Spn- samples (Fig 3A). No genera, not even *Streptococcus*, were found in higher abundance in the Spn+ group. This observation could be explained if lower abundance of *Streptococcus* spp. other than *S*. *pneumoniae* were present in Spn+ samples relative to Spn- samples, as previously reported [27], so the genus of *Streptococcus* as a whole did not exhibit prominence in Spn+ samples. In 12 month-old children, the *Streptococcus* genus was more abundant (p = 8.7 x 10^−14^) and *Corynebacterium* less abundant (p = 9.9 x 10^−5^) in Spn+ samples compared with Spn- samples (Fig 3B). Because Spn colonization is influenced by demograhic factors such as daycare attendance (Table 1), we asked whether daycare attendance affects Spn-induced microbiome change. Indeed, fewer differentially abundant genera were observed between Spn+ and Spn- samples when daycare attendance was considered a covariate: among the genera listed in Fig 3A, only *Dolosigranulum* (p = 0.0007), *Corynebacterium*_1 (p = 0.0003), and *Actinomyces* (p = 0.0007) remained differentially abundant in Spn+ samples versus Spn- samples from 6-month old children (Fig 3C), and only *Corynebacterium*_1 remained differentially abundant in Spn+ samples compared with Spn- samples from 12-month old children (p = 0.041) (Fig 3D).

**Fig 4:**
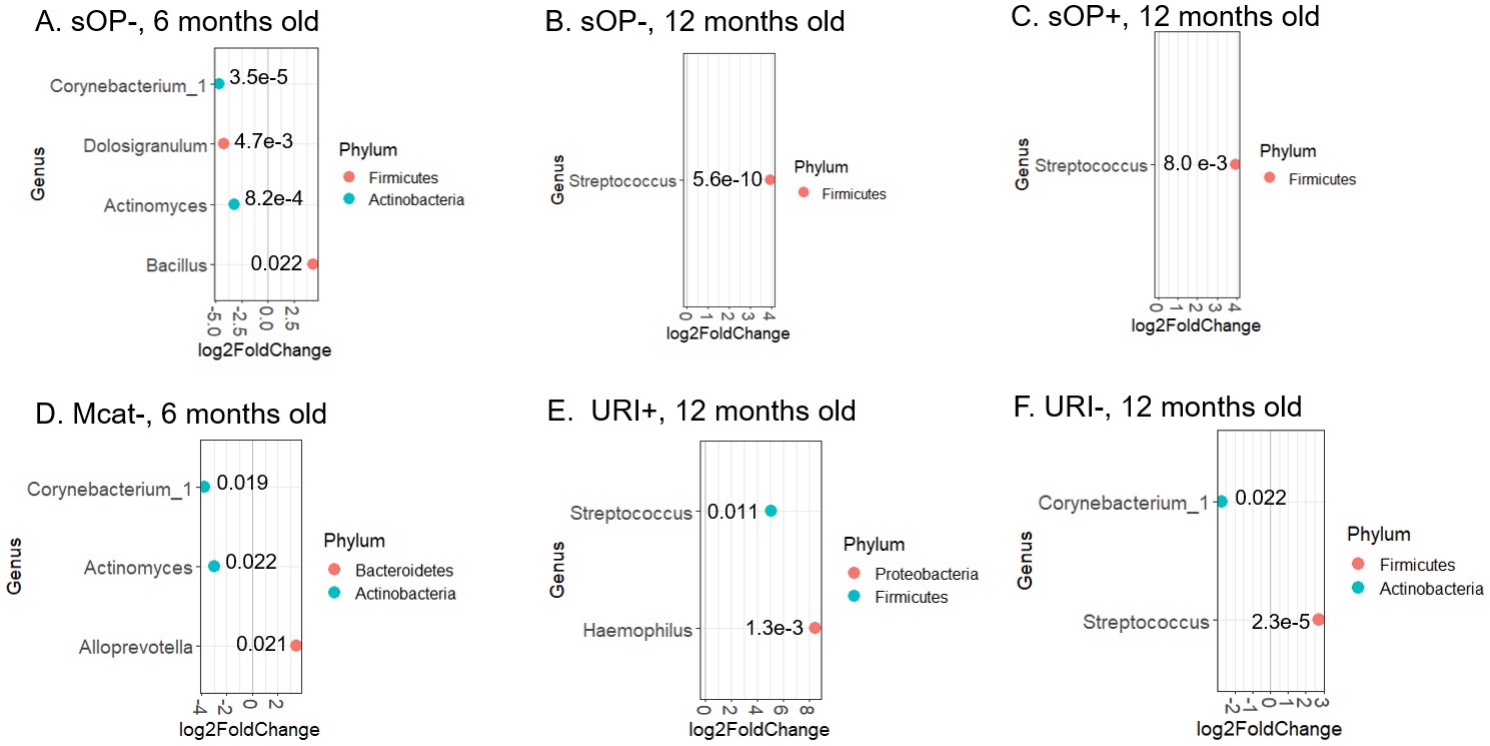
Mcat detection, URI and Otitis Proneness as factors that contribute to microbiome difference between Spn+ and Spn- NP samples. Samples were divided into AOM-free/sOP, Mcat+/Mcat-, and URI+/URI- samples at each age group. Differential abundance of genera between Spn+ and Spn- samples was determined in each subgroup: AOM-free/sOP (A-C), Mcat (D), or URI (E-F), by DESeq2 software (see Materials and methods). The ones with adjusted *p* value less than 0.05 are shown as dots in the graphs. The adjusted *p* value is indicated next to each dot. The x axis exhibits the log_2_ ratio between abundance of bacterial genera in Spn+ samples and that in Spn- samples.

Among other factors associated with Spn colonization, sOP phenotype, Mcat colonization, and URI were significantly associated with Spn colonization in children (Table 2). To determine how these variables influence microbiome changes upon Spn colonization, we divided samples into variable+ and variable- subgroups and analyzed the differential abundance of genera between Spn+ and Spn- samples in each subgroup. We found that in the sOP- subgroup (i.e., AOM-free), *Corynebacterium_1* (p = 3.5 x 10^−5^), *Dolosigranulum* (p = 0.0047), and *Actinomyces* (p = 8.2 x 10^−4^) remained at lower abundance in Spn+ samples relative to Spn- samples, but none of the other genera shown in Fig 3A did, and *Bacillus* became more abundant (p = 0.022) in Spn+ samples from children of 6-month old (Fig 4A). In sOP+ samples, however, no genus was found to differ in abundance between Spn+ and Spn- samples from 6-month old children. These differential findings from sOP+ and sOP- samples indicate that the sOP child phenotype may be associated with significant microbiome changes in response to Spn colonization when children were at 6 months of age. Among children of 12 months old, *Corynebacterium* was no longer at lower abundance but *Streptococcus* stayed at higher abundance in Spn+ samples relative to Spn- samples, once the population was split into AOM-free and sOP+ subgroups (Fig 4B and 4C; p = 5.5 x 10−^10^ and 0.008).

Mcat colonization detected by culture significantly segregated Spn+ and Spn- samples (Table 2) but this only occurred in 6-month old children. Consequently samples from 6-month old children were divided into Mcat+ and Mcat- subgroups to examine the effects of Mcat on microbiome differences when Spn colonization was detected. Within the Mcat- subgroup, *Corynebacterium_1* (p = 0.019) and *Actinomyces* (p = 0.022) were at lower abundance in Spn+ samples, but none of the other genera shown in Fig 3A were, and *Alloprevotella* (p = 0.021) showed higher abundance in Spn+ samples, compared with Spn- samples (Fig 4D). Within the Mcat+ subgroup, however, no genus showed significant difference in abundance between Spn+ and Spn- samples, suggesting that Mcat colonization may override the effects of Spn colonization on microbiome changes.

The third factor we examined was URI, which showed a significant positive association with Spn colonization in children 12 months age but not children 6 months of age (Table 2). Consequently only samples from 12-month old children were divided into URI+ and URI- subgroups and investigated for the influence of URI on microbiome changes in association with Spn colonization. Withiin the URI+ samples, *Haemophilus* (p = 0.0013), in addition to *Streptococcus* (p = 0.011; as shown in Fig 3B), was found at higher abundance in Spn+ samples relative to Spn- samples (Fig 4E). Within the URI- subgroup, *Corynebacterium_1* (p = 0.022) and *Streptococcus* (p = 2.3 x 10^−5^) remained at lower and higher abundance, respectively, in Spn+ samples than in Spn- samples (Fig 4F), as shown in the comparison among all samples from 12-month olds (Fig 3B).

### *Corynebacterium* spp. inhibited Spn proliferation *in vitro* and reduced Spn colonization densities *in vivo*

An inverse relationship between *Corynebacterium* genus and Spn colonization was observed (Fig 3A and 3B), which was not affected by daycare attendance (Fig 3C and 3D) or other pathogens such as *Moraxella* or URI. The non-otitis-prone state was associated with the presence of *Corynebacterium* genus (Fig 4). Therefore, we hypothesized that *Corynebacterium* spp. interfered with Spn NP colonization. To test this, we first isolated *Corynebacterium* spp. from NP samples of children and, using PCR primers that target the *rpoB* gene in *Corynebacterium* [33], we identified *C*. *propinquum* and *C*. *pseudodiphtheriticum* (S2A–S2E Fig).

**Fig 5:**
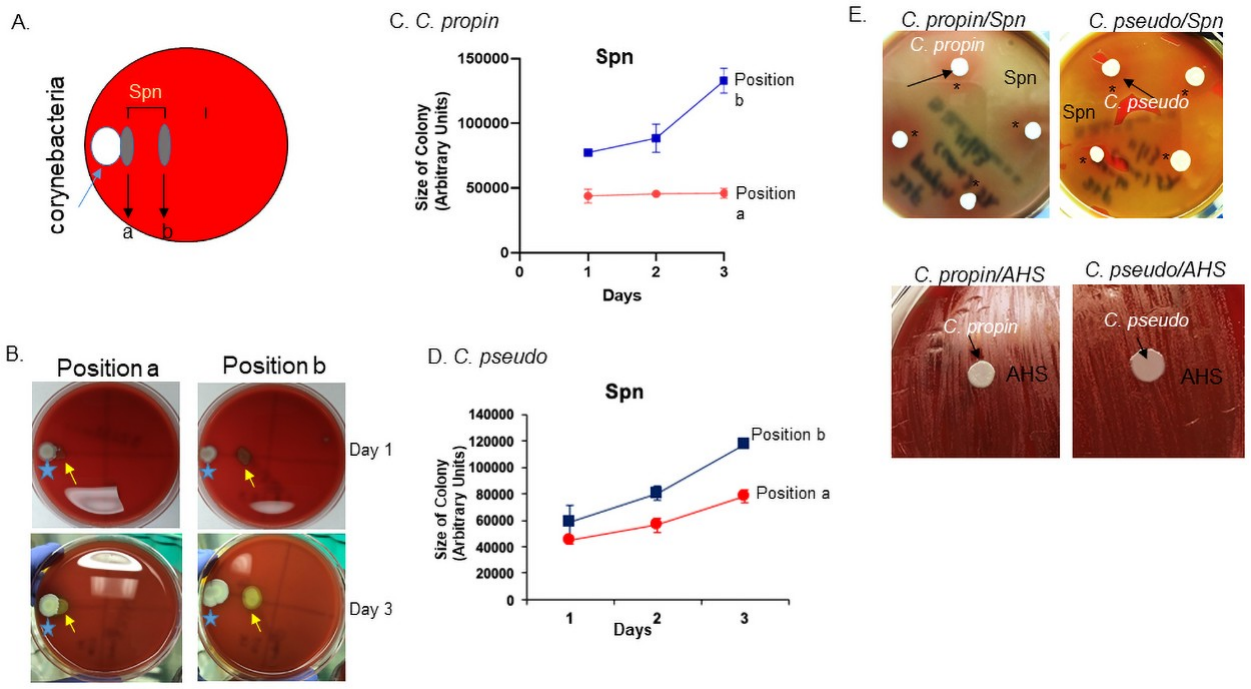
Growth inhibition of *Corynebacterium* by Spn. A) Schematic representation of co-culture experiment for *Corynebacterium* spp. and Spn. Spn22F was inoculated right next to a *Corynebacterium* colony (position a) or with some distance (position b). B) Representative images of *Corynebacterium* spp. co-cultured with Spn22F at position a or b (as shown in A) on day 1 and day 3. C, D) Measurement of size changes in Spn colonies placed at positions a and b next to *C*. *propinquum* (C) or next to *C*. *pseudodiphtheriticum* (D) over 3 days. E) Images of culturing *C*. *propinquum* (left) or *C*. *pseudodiphtheriticum* (right) on top of a lawn of Spn22F (top panels) or AHS (bottom panels).

To evaluate the interaction between *Corynebacterium* and Spn *in vitro*, we inoculated a colony of Spn22F at different distances to *C*. *propinquum* or *C*. *pseudodiphtheriticum* colonies and monitored their growth over three days (Fig 5A and 5B). Spn colonies farther away from the *Corynebacterium* colonies grew faster than those closer (Fig 5C and 5D), indicating an inhibitory effect of *C*. *propinquum* and *C*. *pseudodiphtheriticum* on Spn proliferation. An alternative approach was employed to confirm this inhibitory effect. Concentrated *C*. *propinquum* or *C*. *pseudodiphtheriticum* was added to a lawn of Spn22F on blood agar plates and the growth of Spn22F was visualized the next day. A pink ring (indicatng minimal hemolysis) of Spn22F was observed around *C*. *propinquum* or *C*. *pseudodiphtheriticum* colony (Fig 5E, *), in contrast to the yellow/green lawn (indicating complete hemolysis) of Spn22F, suggesting inhibited growth of Spn22F by *Corynebacterium*. This inhibition was not observed when *C*. *propinquum* or *C*. *pseudodiphtheriticum* was inoculated on a lawn of non-Spn strain of alpha-hemolytic *Streptococcus* (AHS) as a control (Fig 5E) suggesting that the inhibitory effect was not achieved by competition for nutrients in the agar.

## Discussion

The intranasal colonization of Spn is a prerequisite step for its pathogenesis, which occurs via interactions with host as well as other microorganisms in NP [3]. We show here that several genera of bacteria in the NP microbiome correlated negatively or positively with Spn colonization, and some of these correlations appeared to be influenced by daycare attendance or other factors such as upper respiratory infection (URI), *Moraxella* co-colonization, and propensity to develop AOM among young children. Among these genera, *Coryenbacterium* showed a consistent inverse relationship with Spn colonization with little influence by daycare attendance or other factors. We isolated *C*. *propinquum* and *C*. *pseudodiphtheriticum* and found that both inhibited the growth of Spn serotype 22F strain *in vitro*.

We first evaluated the distribution of demographic and other factors that associate with Spn colonization. We found that daycare attendance, Mcat colonization, URI, and AOM recurrence were significantly associated with Spn colonization, but breastfeeding, race, gender, exposure to smoke, symptoms of atopy, presence of siblings were not (Table 1). These findings were generally consistent with previous reports [34–38]. Some inconsistencies were observed, in particular in the effects of race and siblings, which had been reported as risk factors for Spn colonization and infection [37, 39]. This disparity could be due to sampling difference, in that the majority of samples in our study were collected from children in families that were mostly middle class and caucacian. We did not observe influence of antibiotic treatment on colonization of Spn, inconsistent with what was reported [37]. Perhaps the antibiotic regimens (types of antibiotics, duration and intervals) in our cohorts differ from the previous report.

Microbiota in NP consists of pathogens and commensals [4–6, 45], both of which influence Spn colonization and pathogenesis [3, 45, 46]. We focused on the roles of commensals on Spn colonization, because they are relatively less studied and impose novel therapeutic potentials for treating Spn-related diseases. The Actinobacteria phyla had an inverse relationship with Spn colonization in our chorts, consistent with previous reports on microbiome distribution in human nostrils [47]. A dominant genus of Actinobacteria phylum in nostrils is the *Corynebacterium* [48], which was reported to negatively correlate with Spn colonization in pediatric samples [15, 17, 18] and was confirmed in our study (Fig 3). In URI+ samples however, *Corynebacterium* no longer exhibited inverse relationship with Spn colonization. Perhaps, the condition of URI supports the colonization of *Corynebacterium*, counteracting the effects of Spn. This notion is consistent with the report by Edouard et al that *C*. *propinquum* was elevated in patients with symptoms of viral respiratory tract infections, compared with healthy controls [49].

We also found an inverse relationship between *Dolosigranulum* and Spn, as previously reported [18]. Our study revealed a few new taxa that correlated negatively with Spn colonization in NP of 6-month olds—*Actinomyces*, *Prevotella*, *Veillonella*, *Gemella*, and *Anoxybacillus*. Among these, *Prevotella*, *Veillonella*, *and Gemella* were found inversely correlated with pneumonia in adults and elderly people [50], who had elevated Spn in oropharyngeal samples. *Prevetella* was reported to correlate with a reduced risk of hospital-acquired pneumonia in ICU patients [51]. Notably, these anaerobic genera were most pronouncedly influenced by daycare attendance and other confounding factors (Figs 3C and 4), suggesting their sensitivity to these factors. They were no longer found differentially abundant between the Spn+ and Spn- NPs of 12-month old children (Fig 3B). Perhaps nasal microbiome in young children is more susceptible to changes induced by Spn colonization than older children. It is known that microbiome composition undergoes profound changes within the first year of a child’s life [50, 52], with bacteria density increasing and gained dominance of certain taxa such as *Moraxella* spp. Spn colonization may thus not be sufficient to alter the abundance of taxa like *Moraxella* in the NP of 12-month olds. In contrast, the abundance of *Corynebacterium* spp. decreases as a child ages, making them remain susceptible to depletion upon Spn colonization at the age of 12 months, as was observed (Fig 3B).

Among human commensals, the *Corynebacterium* is one of the most studied and has been extensively reported to correlate with a healthy state in nasopharynx [53–59]. Its function in Spn pathology was also reported. *C*. *accolens*, for example, was found to inhibit Spn *in vitro* via releasing fatty acid; its functions *in viv*o however were not reported [15]. On the other hand, *C*. *pseudodiphtheriticum* 090104 was shown to inhibit secondary infection of *S*. *pneumoniae in viv*o probably by inducing elevated TNF-alpha and interferon gamma levels [19]. The immune effect of *Corynebacterium* was reported to be strain-specific, since a different *C*. *pseudodiphtheriticum* strain did not exhibit this function [20].

Our study showed the *in vitro* effects of two *Corynebacterium* species, *C*. *propinquum* and *C*. *pseudodiphtheriticum*, on Spn proliferation. We do not know the nature of this inhibition, but we suspect that it would differ from aforementioned inhibition by *C*. *accolens* [15], since *C*. *accolens* is a lipophilic species and requires lipid for its growth but *C*. *propinquum* and *C*. *pseudodiphtheriticum* are not [13]. It was reported recently that *C*. *propinquum* released siderophores to inhibit the growth of coagulase-negative *Staphylococcus* species but not coagulase-positive *Staphylococcus* species such as *S*. *aureus* [60]. This inhibition was not observed from *C*. *pseudodiphtheriticum* so likely differs from the inhibition we observed in this study.

Our study has several limitations. First, 16S rRNA gene sequencing at the V4 region does not provide resolution to the species level, therefore we do not know what bacterial species correlate with Spn colonization. This limitation significantly reduces the number of taxa to be uncovered and imposes difficulty in isolating relevant commensals for follow-up studies. Full-length 16S rRNA gene sequencing or shotgun metagenomics with genome reconstruction would need to be performed to overcome this shortcoming. Second, our *in vitro* assays imply that the *Corynebacterium* species inhibit the proliferation of Spn22F. This interpretation needs to be considered cautiously, as only one isolate of each species was tested in our study. Additionally, the *in vitro* effects we observed may not directly translate to their effects on Spn colonization in patients, since Spn colonization involves more steps (survival, adhesion, etc.) than proliferation. Furthermore, we only used one Spn strain (Spn22F) in our study whereas in human more than 90 strains of Spn have been identified. More Spn strains would need to be tested to evaluate whether the *Corynebacterium* species inhibit Spn proliferation in general, but not specific to Spn22F. We also do not know whether the inhibitory effects of *Corynebacterium in vitro* could be translated to their effects *in vivo*. *C*. *pseudodiphtheriticum* had been shown recently to induce a pro-immune response in mice [19], which in turn may imped the colonization of Spn. In contrast, *C*. *propinquum* was reported to correlate with anti-flammatory mediators in human nasopharynx [61]. Whether and what type of immune responses the *Corynebacterium* species may elicit *in vivo* to imped Spn colonization thus await future investigation. Finally, our study population is from middle-class, suburban pediatric practices and thus may not represent the general population which are more socioeconomically diverse.

## Supporting information

S1 Fig Alpha diversity does not differ between Spn+ and Spn- NP samples. Alpha diversity indices of nasal microbiome from Spn+ or Spn- children of 6 and 12 months olds were calculated and graphed in box plots.
(TIF)

S2 Fig Isolation of *Corynebacterium propinquum* and *Corynebacterium pseudodiphtheriticum*. A) Agarose gel electrophoresis of PCR products from *rpoB* gene. Lanes 1 and 3: PCR products from colonies grown on chocolate plates; lanes 2 and 4: PCR products from colonies grown on blood agar plates. The growth of *Corynebacterium* on chocolate plates was not as robust as on blood agar plates, so the colonies grown on chocolate plates were found to differ from *Corynebacterium* and served as a negative control. B,C) Sequences of PCR products from two *Corynebacterium* species that were later identified as *C*. *propinquum* and *C*. *pseudodiphtheriticum*. D) Alignment of PCR sequences from *C*. *propinquum* against GeneBank database. E) Alignment of PCR sequences from *C*. *pseudodiphtheriticum* against GenBank database.
(TIF)

S1 Table Demorgraphic and clinical information of the samples. (XLSX)

S1 Raw images (TIF)
