# Supplementary figures and images for: Nasopharyngeal microbiome composition associated with Streptococcus pneumoniae colonization suggests a protective role of Corynebacterium in young children
Source: PLoS One. 2021 Sep 16;16(9):e0257207. doi: 10.1371/journal.pone.0257207 (PMC8445455; doi:10.1371/journal.pone.0257207)

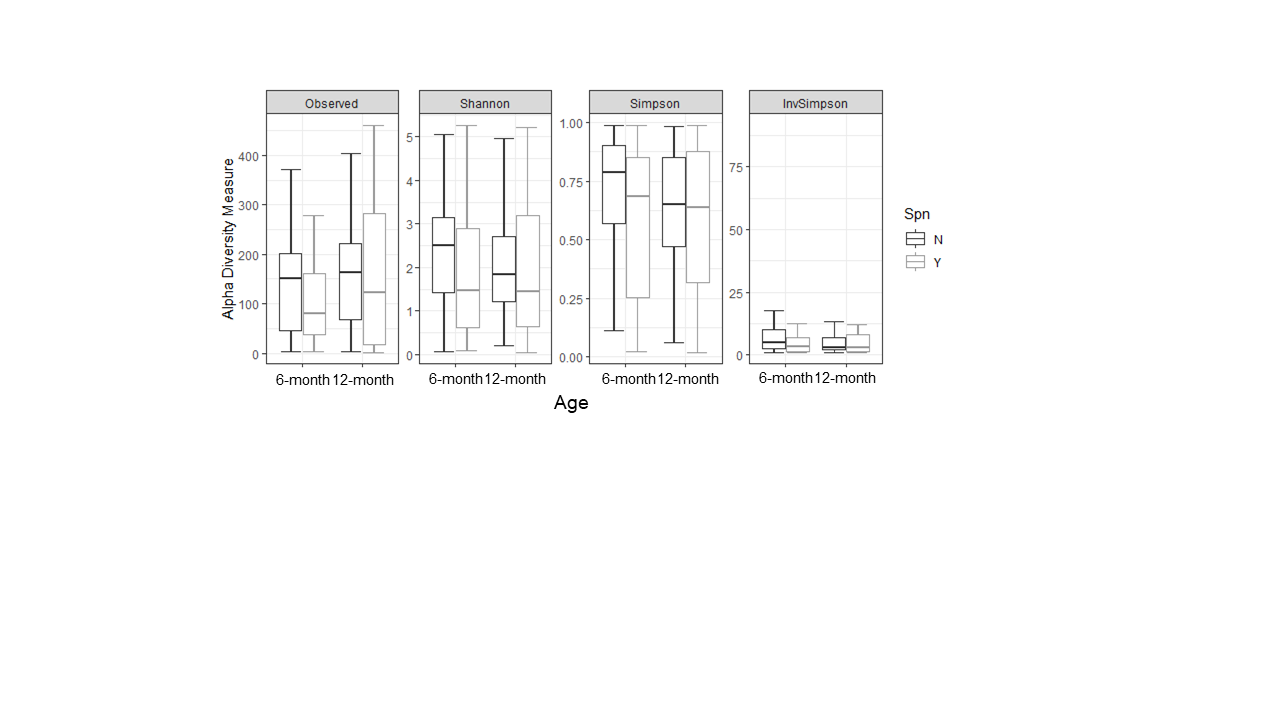

Supplement: S1 Fig — Alpha diversity indices of nasal microbiome from Spn+ or Spn- children of 6 and 12 months olds were calculated and graphed in box plots. (TIF) [file pone.0257207.s001.tif]

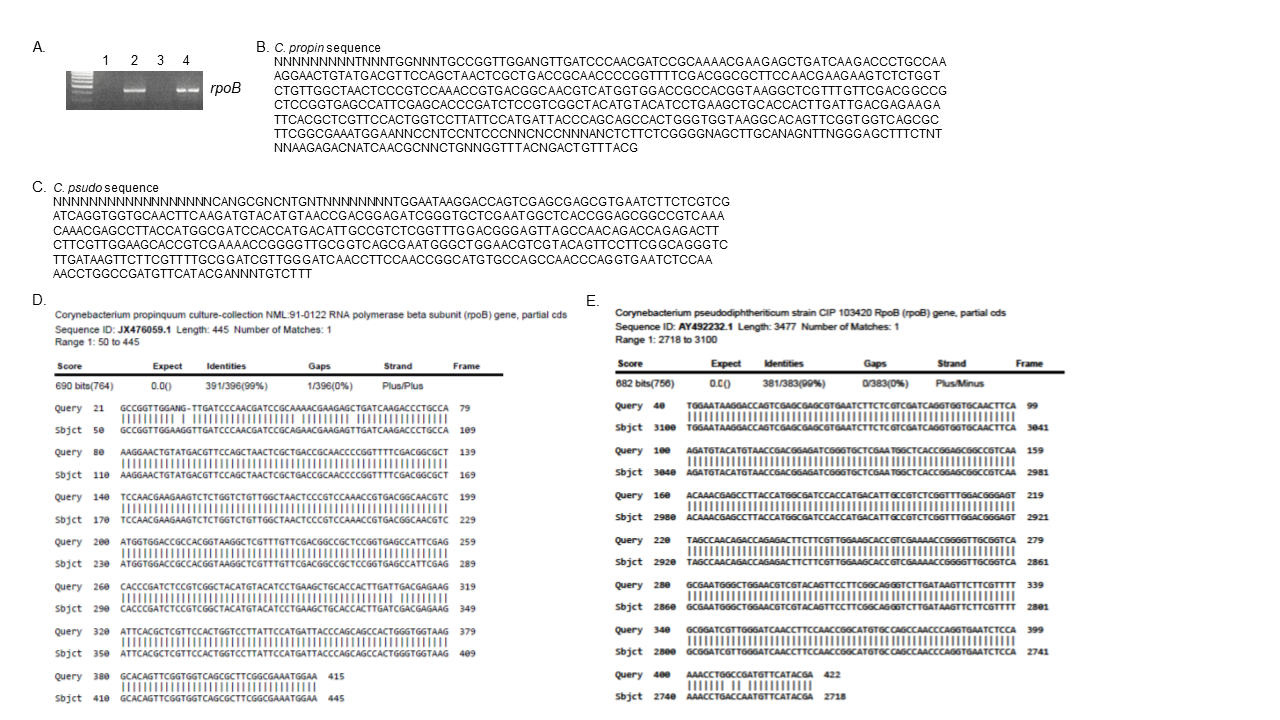

Supplement: S2 Fig — A) Agarose gel electrophoresis of PCR products from rpoB gene. Lanes 1 and 3: PCR products from colonies grown on chocolate plates; lanes 2 and 4: PCR products from colonies grown on blood agar plates. The growth of Corynebacterium on chocolate plates was not as robust as on blood agar plates, so the colonies grown on chocolate plates were found to differ from Corynebacterium and served as a negative control. B,C) Sequences of PCR products from two Corynebacterium species that were later identified as C. propinquum and C. pseudodiphtheriticum. D) Alignment of PCR sequences from C. propinquum against GeneBank database. E) Alignment of PCR sequences from C. pseudodiphtheriticum against GenBank database. (TIF) [file pone.0257207.s002.tif]
